# Potential thresholds of critically increased cardiac-related spinal cord motion in degenerative cervical myelopathy

**DOI:** 10.3389/fneur.2024.1411182

**Published:** 2024-06-24

**Authors:** Nikolai Pfender, Catherine R. Jutzeler, Michèle Hubli, Paulina S. Scheuren, Dario Pfyffer, Carl M. Zipser, Jan Rosner, Susanne Friedl, Reto Sutter, José M. Spirig, Michael Betz, Martin Schubert, Maryam Seif, Patrick Freund, Mazda Farshad, Armin Curt, Markus Hupp

**Affiliations:** ^1^Spinal Cord Injury Center, Balgrist University Hospital, University of Zurich, Zurich, Switzerland; ^2^Department of Health Sciences and Technology, ETH, Zurich, Zurich, Switzerland; ^3^International Collaboration on Repair Discoveries (ICORD), Faculty of Medicine, University of British Columbia, Vancouver, BC, Canada; ^4^School of Medicine, Department of Anesthesiology, Perioperative and Pain Medicine, Stanford University, Palo Alto, CA, United States; ^5^Department of Neurology, Bern University Hospital, Inselspital, University of Bern, Bern, Switzerland; ^6^Department of Radiology, Balgrist University Hospital, University of Zurich, Zurich, Switzerland; ^7^University Spine Center Zurich, Balgrist University Hospital, University of Zurich, Zurich, Switzerland; ^8^Department of Neurophysics, Max Planck Institute for Human Cognitive and Brain Sciences, Leipzig, Germany

**Keywords:** cervical cord, spinal cord motion, degenerative cervical myelopathy (DCM), cervical spondylotic myelopathy (CSM), phase contrast MRI (PC-MRI), spinal stenosis, spinal cord oscillations

## Abstract

**Introduction:**

New diagnostic techniques are a substantial research focus in degenerative cervical myelopathy (DCM). This cross-sectional study determined the significance of cardiac-related spinal cord motion and the extent of spinal stenosis as indicators of mechanical strain on the cord.

**Methods:**

Eighty-four DCM patients underwent MRI/clinical assessments and were classified as MRI+ [T2-weighted (T2w) hyperintense lesion in MRI] or MRI− (no T2w-hyperintense lesion). Cord motion (displacement assessed by phase-contrast MRI) and spinal stenosis [adapted spinal canal occupation ratio (aSCOR)] were related to neurological (sensory/motor) and neurophysiological readouts [contact heat evoked potentials (CHEPs)] by receiver operating characteristic (ROC) analysis.

**Results:**

MRI+ patients (*N* = 31; 36.9%) were more impaired compared to MRI− patients (*N* = 53; 63.1%) based on the modified Japanese Orthopedic Association (mJOA) subscores for upper {MRI+ [median (Interquartile range)]: 4 (4–5); MRI−: 5 (5–5); *p* < 0.01} and lower extremity [MRI+: 6 (6–7); MRI−: 7 (6–7); *p* = 0.03] motor dysfunction and the monofilament score [MRI+: 21 (18–23); MRI−: 24 (22*-*24); *p* < 0.01]. Both patient groups showed similar extent of cord motion and stenosis. Only in the MRI− group displacement identified patients with pathologic assessments [trunk/lower extremity pin prick score (T/LEPP): AUC = 0.67, *p* = 0.03; CHEPs: AUC = 0.73, *p* = 0.01]. Cord motion thresholds: T/LEPP: 1.67 mm (sensitivity 84.6%, specificity 52.5%); CHEPs: 1.96 mm (sensitivity 83.3%, specificity 65.6%). The aSCOR failed to show any relation to the clinical assessments.

**Discussion:**

These findings affirm cord motion measurements as a promising additional biomarker to improve the clinical workup and to enable timely surgical treatment particularly in MRI− DCM patients.

**Clinical trial registration:**

www.clinicaltrials.gov, NCT 02170155.

## 1 Introduction

Degenerative changes of the cervical spine may result in cervical spinal stenosis with consecutive spinal cord compression leading to the clinical syndrome of degenerative cervical myelopathy (DCM) (1). DCM is the most common cause of non-traumatic incomplete spinal cord injury (1). The underlying pathophysiology of DCM is attributed to immediate (i.e., direct or static) cord compression, spinal malalignment leading to altered cord tension, impaired vascular supply, and repeated dynamic injury (2–4). Cervical MRI is deemed essential for diagnosing DCM (5). However, the extent of spinal canal stenosis (as a proxy of static cord compression) insufficiently explains the disease severity and progression (6, 7). Numerous anatomic readouts have been investigated to reflect the severity of compression in individuals with spinal stenosis (8–10), but only modest correlations to the patient’s functional status could be observed (11, 12). Quantitative dimensional measures of spinal stenosis are confounded by the diversity of configurations [e.g., central vs. lateral compression; anterior–posterior and lateral compression simultaneously; inter-individual variance of spinal cord cross sectional area (CSA)]. Recently, the adapted spinal canal occupation ratio (aSCOR), a ratio between the segmental spinal cord CSA and the spinal canal CSA, has been introduced (13) to assess the extent of individual spinal cord compression at a segmental stenosis. In addition to the severity of cord compression, dynamic spinal cord injury primarily due to cardiac related cranio-caudal oscillations emerged as a potential contributing factor within the DCM pathophysiology, which can be assessed using phase contrast MRI (14–17). Cranio-caudal directed motion was shown to be the cardinal change in DCM patients (18). Interestingly, the motion pattern of the spinal cord in most DCM patients lacks a physiological resting phase within the cardiac cycle (14), ending in enduring (“restless”) oscillations. Additionally, increased spinal cord oscillations at the site of a spinal stenosis contribute to stretch and compression of cord tissue in adjacent segments (19). These pathophysiological changes caused intensified dynamic mechanical burden to the spinal cord in a computational model (20), which could be hypothesized to contribute to subsequent tissue damage, causing neurological deterioration. The heart beats approximately 100′000 times per day (i.e., 70 beats per minute), resulting in 100′000 spinal cord oscillations per day. Given the frequent oscillations, dynamic spinal cord strain due to a local stenosis may play a significant pathophysiological role in DCM. Underlining this assumption, increased spinal cord oscillations at the level of cervical spinal stenosis (14–17) were shown to be associated with upper limb dysesthesia (15), impaired sensory evoked potentials (16) and decreased sensory scores (i.e., light touch sensation) (17). While increased spinal cord motion was reported to correlate with narrowed anatomic conditions (17, 19, 21), its added diagnostic value in DCM patients remains to be demonstrated. Interestingly, patients with a comparable anatomical situation (i.e., comparable extent of spinal stenosis in MRI) can present with absolutely divergent motion patterns (i.e., only moderate versus extensively increased oscillations). This discrepancy points to a possible value of spinal cord motion measurements as a proxy for dynamic mechanical strain as an additional biomarker.

This study aimed to investigate the diagnostic value of either spinal cord motion (i.e., cranio-caudal displacement) or anatomical measures of the severity of a spinal canal stenosis (i.e., aSCOR) to identify DCM patients with pathologic neurological (i.e., light touch, pin prick, and monofilament sensation; strength) and neurophysiological [i.e., contact heat evoked potentials (CHEPS)] assessments. While DCM patients with T2w hyperintense lesions in MRI [i.e., edema; signs of established myelopathy like cystic lesions, snake eyes (MRI+)] show poorer outcomes after decompression (9), complementary diagnostic methods are particularly needed in patients without potentially irreversible cord damage [i.e., without T2w hyperintense lesions in MRI (MRI−)]. We hypothesized, that increased spinal cord motion can already be observed in MRI− patients and may identify patients with pathologic neurological and neurophysiological assessments especially in this patient group.

## 2 Materials and methods

### 2.1 Study design and population

Prospective, cross-sectional study. Between October 2016 and December 2022, in total, 147 DCM patients were recruited in the outpatient clinic of the Balgrist University Hospital, Zurich. Nine of these patients did not participate in an MRI measurement (due to: claustrophobia; body weight exceeded the limits for the MRI Scanner; withdrew their consent; excluded due to comorbidities). Of the remaining 138 patients, 91 had completely available clinical, neurological and neurophysiological assessments for this analysis. Seven patients were excluded due to previous spine surgery. Finally, 84 patients were included in this analysis. A subset of this patient cohort has been reported previously (14, 22), however, the findings presented in this study are novel. Inclusion criteria: cervical spinal stenosis on the T2-weighted (T2-w) MRI; clinical symptoms consistent with DCM (23) (i.e., pain, sensory or motor deterioration in the upper or lower limbs, gait or bladder dysfunction); age 18–80 years. Patients suffering from a competing neurological disease with potential bias to clinical and neurophysiological assessments were excluded. Additional exclusion criteria: MRI contraindications, epileptic seizures, mental illness, severe medical illness and pregnancy. Study data were collected and managed using REDCap electronic data capture tools hosted at Balgrist University Hospital, Zurich, Switzerland.

### 2.2 Clinical and neurological assessments

Symptom severity and functional impairment were assessed with the modified Japanese Orthopedic Association (mJOA) score (24). The mJOA score assesses upper and lower extremity motor, upper extremity sensory, and bladder function and ranges from 0 to 18, where lower scores reflect a higher symptom burden. A standardized neurological examination was performed according to the International Standards for Neurological Classification of Spinal Cord Injury including segmental sensory assessments and motor scores (25). Pinprick and light touch sensations were tested at dermatomes C4-S2 and classified on a scale of 0–2 (0 = absent, 1 = impaired, 2 = normal). Strength was classified in the myotomes C5-Th1 (upper extremities) and L2-S1 (lower extremities) according to the Medical Research Council Scale for Muscle Strength from 0 to 5 (0 = no contraction; 1 = flicker or trace of contraction; 2 = active movement, with gravity eliminated; 3 = active movement, against gravity; 4 = active movement, against gravity and resistance; 5 = normal power). Sensory scores were calculated for the upper extremity dermatomes (C4 to T1) and for the dermatomes of the trunk/lower extremities (T2 to S2). Maximum sensory scores are 24 points each for the upper extremity light touch (UELT) and pin-prick (UEPP) scores and 72 points for the trunk/lower extremities light touch (T/LELT) and pin prick (T/LEPP) scores, respectively. Maximum motor scores are 50 points for the upper extremity (UEMS) and lower extremity (LEMS) motor score. Sensibility of the hand was additionally evaluated with the respective subset of the Graded and Redefined Assessment of Strength and Prehension (GRASSP) for hand function (26), including upper extremity monofilament (UEMF) perception in the dermatomes C6, C7, and C8 (each dorsum of the hand) with a maximum score of 4 each dermatome per side (maximum score of 24 points).

### 2.3 Neurophysiological assessments

Contact heat evoked potentials (CHEPs) were recorded as they have been proven to be most sensitive to reveal central spinal cord damage in DCM (27). Segmental CHEPs were recorded after stimulation of the dermatome C6 (in patients with maximum stenosis located at segment C3 and C4) or C8 (in patients with maximum stenosis located at segment C5 and C6) of the clinically more affected side. Below-level CHEPs were recorded after stimulation of the dermatome T4 of the clinically more affected side. We used the CHEPs thermode (Pathway, Medoc, RamatYishi, Israel) to apply 15–20 contact-heat stimuli (baseline 42°C; destination 52°C; 70°C/s; interstimulus interval 8–12 s) (28). The thermode was repositioned after each stimulus to avoid peripheral fatigue and habituation. The recording set-up for electroencephalographic signals was the same as performed in previous studies (27, 29). Briefly, CHEPs were recorded from the vertex (Cz in a 10–20 electrode configuration referenced to A1, A2) using subdermal needle electrodes (12 mm; Spes Medica s.r.l., Battipaglia, Italy). All signals were sampled at 2,000 Hz using a preamplifier (20,000x, ALEA Solutions, Zurich, Switzerland) and bandpass-filtered in the range of 0.5–30 Hz. The recording window was set at 0.5 s pre-trigger and 1 s post-trigger in a customized program based on LabView (V2.6.1. CHEP, ALEA30 Solutions, Zurich, Switzerland). Signals contaminated with artifacts were excluded. Additional stimuli were applied as necessary to generate a total averaged EP of 15 artifact-free signals. Each averaged N/P waveform was evaluated by two independent raters (MiH, JR, PS) and classified as (1) abolished, (2) impaired, or (3) normal. In case no N/P waveform was detectable, the response was classified as abolished. The classifications “impaired” and “normal” were defined based on the N2-latency of the waveforms. If the N-latency in response to stimulation was within the normal range (mean + 2SD) (29), the response was classified as “normal.” If the N-latency was outside of this range, the response was classified as “impaired.”

### 2.4 MRI measurements

All patients underwent an MRI protocol including sagittal and axial T2-weighted (T2-w) MRI using a 3 Tesla MRI scanner (MAGNETOM SkyraFit or MAGNETOM Prisma; Siemens Healthcare, Germany, Erlangen). Cranio-caudal spinal cord motion was assessed with axial and sagittal (from August 2018) phase contrast (PC)-MRI as described previously (14, 21, 22). The measurements were retrospectively cardiac gated, using a pulsoxymeter and 128 measurements were taken per cardiac cycle. Total acquisition time for the MRI protocol was approximately 23 min (MRI parameters are listed in Supplementary Table 1). Image processing was performed using an open-source DICOM viewer.^1^ PC-MRI assessed spinal cord motion at the most stenotic cervical segment (upon visual inspection in T2-w MRI) and was visually checked for artifacts prior to image processing. The spinal cord motion readout was cord displacement, reflecting the overall cord motion during the cardiac cycle (i.e., area under the curve of the velocity signal after phase drift correction). Phase drift correction and displacement calculation was done as reported previously (14, 21). Shortly, velocity signal was assessed within 20 time points during the cardiac cycle. Velocity was encoded in gray values, while darker colors encoded caudal and brighter colors cranial velocities. The mean of the measured greyscale values within the region of interest (placed into the spinal cord at the intervertebral disk level) in each of 20 timepoints during one cardiac cycle was used for calculation of the velocity. Velocity data were corrected for phase drift prior further statistical analysis. Displacement values were calculated by stepwise summation of calculated squared areas (time*velocity). Negative velocity values were transformed to a positive value for calculation of the area under curve. In segments with no available axial PC-MRI (i.e., missing measurement or artifact), the sagittal PC-MRI displacement value was used if available. A good agreement between axial and sagittal PC-MRI cord motion measurements in DCM was previously reported (25). The presence of a hyperintense T2-w lesion (e.g., diffuse T2 hyperintensity, cystic lesions, and snake eyes) within the spinal cord (MRI+ patients) was visually evaluated in axial and sagittal T2-w MRI (MH). In axial T2-w MRI, the cross-sectional area (CSA) of the spinal cord and the spinal canal were manually measured. To reflect the severity of the spinal canal stenosis (a proxy of static cord compression), the aSCOR was calculated [aSCOR (%) = spinal cord CSA divided by spinal canal CSA and multiplied by 100] (13).

### 2.5 Statistical analysis

Statistical analysis was conducted using SPSS (IBM SPSS Statistics for Windows, Version 29.0; Armonk, NY; IBM Corp). Shapiro–Wilk test was used to test data for normal distribution. While most variables were not normally distributed, group median and interquartile range (IQR) are reported. Differences between patient groups with (MRI+) and without (MRI−) T2w hyperintense lesion in MRI were calculated with the Mann–Whitney U test (i.e., age, UEMS, UELT, UEPP, UEMF, LEMS, T/LELT, T/LEPP, aSCOR, and displacement) and Chi^2^, respectively, Fishers exact test (i.e., sex, mJOA score, and sub scores, proportion of pathologic assessments, proportion of patients with a multi-segmental stenosis, and number of stenotic segments). Receiver operating characteristic (ROC) curve analysis (30) was applied to test the diagnostic value of anatomic (i.e., aSCOR) and spinal cord motion (i.e., displacement) readouts as predictors to identify patients with pathologic neurological and neurophysiological assessments. ROC analysis was conducted separately in MRI+ and MRI− subgroups. For ROC analysis, neurological and neurophysiological assessments (i.e., outcome variables) were dichotomized to normal (i.e., maximum score of neurological assessment, respectively, normal neurophysiological examination) and pathologic (i.e., at least one point below the maximum of the neurological assessment, respectively, impaired or abolished neurophysiological examination). The area under the receiver operating characteristics curve (ROC-AUC) was estimated for the displacement and aSCOR values as predictors for the corresponding outcome parameters: UELT; UEPP; UEMF; UEMS; T/LELT; T/LEPP; LEMS; segmental CHEPS; below-level CHEPs. LEMS were excluded from ROC analysis due to low prevalence of pathologic assessments (MRI+ patients: 12.9%; MRI− patients. 3.8%). Statistical significance was set at *α* < 0.05.

## 3 Results

### 3.1 Demographics and number of available datasets

Of 84 enrolled patients, 31 (36.9%) presented with (MRI+ group) and 53 (63.1%) presented without (MRI−) T2w hyperintense lesion within the spinal cord. The MRI+ and MRI− groups did not differ in age or sex (Table 1A). The monofilament score was missing in five (9.4%) MRI− and two (6.5%) MRI+ patients, while all other assessments were available in all patients.

**Table 1:** Basic demographics, clinical/neurological assessments, and MRI findings in patient groups with and without T2w hyperintense lesion in MRI.

			MRI+ patients		MRI− patients		
			*N*		*N*		*p*
**A. Demographics**							
Sex (female) [*N* (%)]			31	7 (22.6%)	53	20 (37.7%)	0.23
Age (years) [median (IQR)]			31	60 (52–65.5)	53	54 (46–61)	0.09
**B. Clinical assessments**							
mJOA total score (max. 18) [median (IQR)]			31	15 (14–17)	53	17 (16–17)	0.08
mJOA subscores	UE motor dysfunction (max. 5) [median (IQR)]		31	4 (4–5)	53	5 (5–5)	**<0.01**
	UE sensory dysfunction (max. 3) [median (IQR)]		31	2 (2–3)	53	2 (2–3)	0.26
	LE motor dysfunction (max. 7) [median (IQR)]		31	6 (6–7)	53	7 (6–7)	**0.03**
	bladder dysfunction (max. 3) [median (IQR)]		31	3 (2–3)	53	3 (2–3)	0.55
**C. Neurological assessments**							
UE motor score (max. 50) [median (IQR)]			31	50 (49–50)	53	50 (50–50)	0.69
UE light touch score (max. 24) [median (IQR)]			31	24 (20.5–24)	53	24 (21–24)	0.68
UE pin prick score (max. 24) [median (IQR)]			31	23 (18.5–24)	53	23 (20–24)	0.45
UE monofilament score (max. 24) [median (IQR)]			29	21 (18–23)	48	24 (22–24)	**<0.01**
LE motor score (max. 50) [median (IQR)]			31	50 (50–50)	53	50 (50–50)	0.14
T/LE light touch score (max. 72) [median (IQR)]			31	72 (72–72)	53	72 (72–72)	0.84
T/LE pin prick score (max. 72) [median (IQR)]			31	72 (72–72)	53	72 (72–72)	0.83
**D. MRI findings**							
Multisegmental stenosis [*N* (%)]			31	21 (67.7%)	53	34 (64.2%)	0.82
Number of stenotic segments [median (IQR)]			31	2 (2)	53	2 (1–2)	0.70
Maximum stenosis		Segment [*N* (%)]					
		C3/4		10 (32.3%)		3 (5.7%)	**0.01**
		C4/5		5 (16.1%)		14 (26.4%)	
		C5/6		15 (48.4%)		34 (64.2%)	
		C6/7		1 (3.2%)		2 (3.8%)	

MRI−, Patients without T2w hyperintensity lesion in MRI; MRI +, Patients with T2w hyperintensity lesion in MRI; *N*, Number of datasets; mJOA, Modified Japanese Orthopedics Association score; UE, Upper extremities; LE, Lower extremities; IQR, Interquartile range; T/LE, trunk/lower extremity.

### 3.2 Clinical and neurological assessments

**Figure 1:**
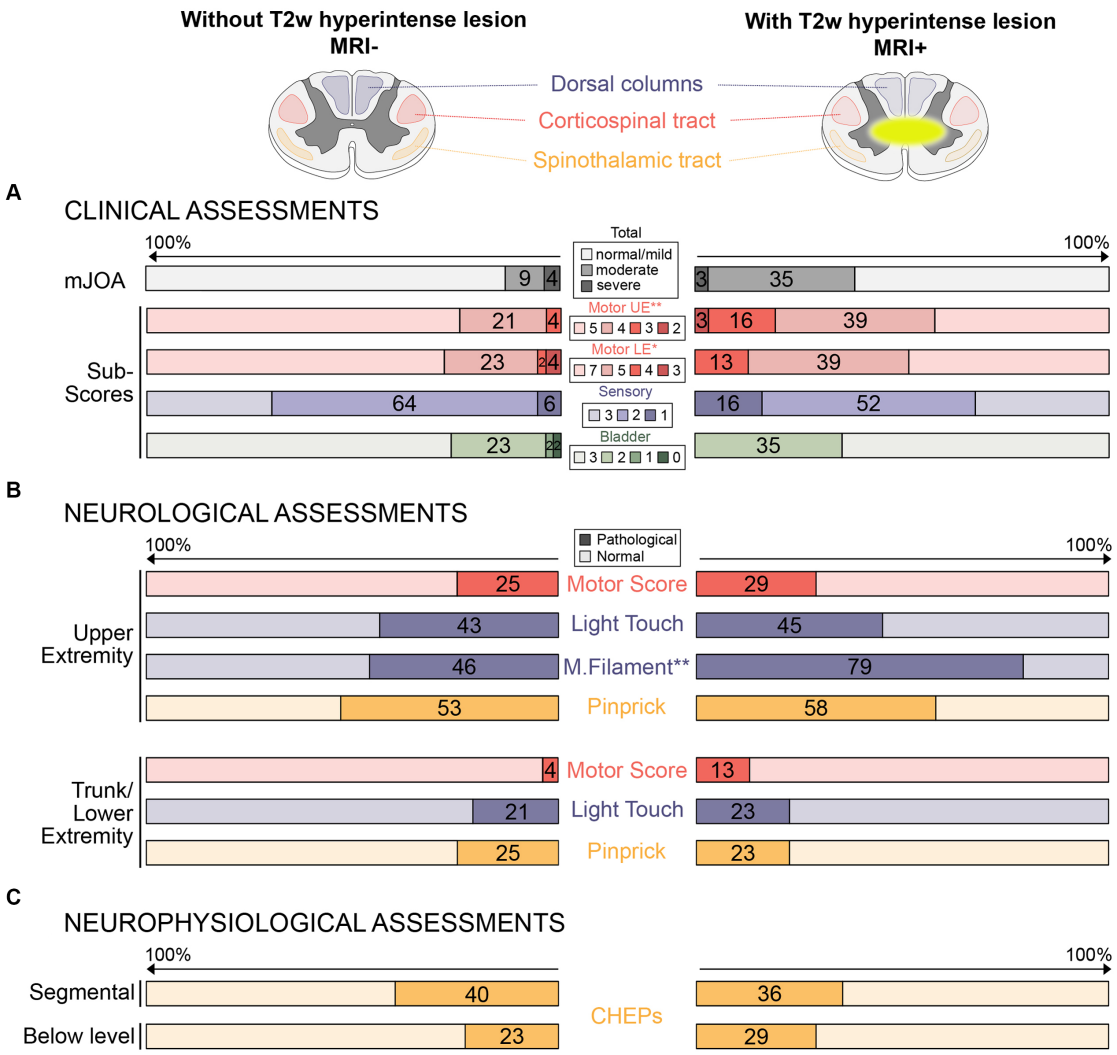
Proportions of pathological clinical, neurological, and neurophysiological assessments in patients without and with T2w hyperintense lesion in MRI. **(A)** Proportions (%) of pathological clinical assessments (i.e., total mJOA and mJOA subscores) are illustrated in patients without (left side) and with (right side) T2w hyperintense lesion in MRI. **(B)** Proportions (%) of pathological neurological assessments (i.e., upper and lower extremity motor and sensor function) are shown for both groups. **(C)** Proportions (%) of pathological neurophysiological assessments (i.e., segmental and below level contact heat evoked potentials) are shown for both groups. Red colors represent motor assessments (i.e., integrity of the corticospinal tract), blue colors represent large fiber sensory assessments (i.e., integrity of dorsal columns), and yellow colors represent small fiber sensory assessments (i.e., integrity of the spinothalamic tract). The darker colors represent lower scores/pathological assessments (i.e., more impairments). Differences between groups (^*^p < 0.05; ^**^p < 0.01) was shown for the mJOA subscores for upper and lower extremity motor function **(A)** and monofilament sensation **(B)**, but not for other assessments. mJOA, modified Japanese Orthopedics Association score; UE, Upper extremity; LE, Lower extremity; CHEPs, Contact heat evoked potentials; and M.filament, monofilament.

The MRI+ group had lower mJOA subscores for UE and LE motor dysfunction (Table 1B; Figure 1A). No differences between MRI+ and MRI− groups were observed for the total mJOA score and the mJOA subscores for upper extremity sensory and bladder dysfunction. MRI+ patients presented with lower monofilament scores, while no differences between MRI+ and MRI− groups could be observed for other sensory or motor assessments (Table 1C). Pathologic neurological assessments were primarily found at upper extremities and sensory deficits were more prevalent than motor deficits in both patient groups (Figure 1B). In MRI+ patients, the proportion of pathologic MF testing was higher compared to MRI− patients, while no differences between MRI+ and MRI− patient groups were observed for other assessments (Figure 1B).

### 3.3 Neurophysiological assessments

The proportion of pathologic neurophysiological measurements was similar in MRI+ and MRI− patients for segmental and below-level CHEPS (Figure 1C).

### 3.4 Localization and extent of cervical spinal stenosis

**Figure 2:**
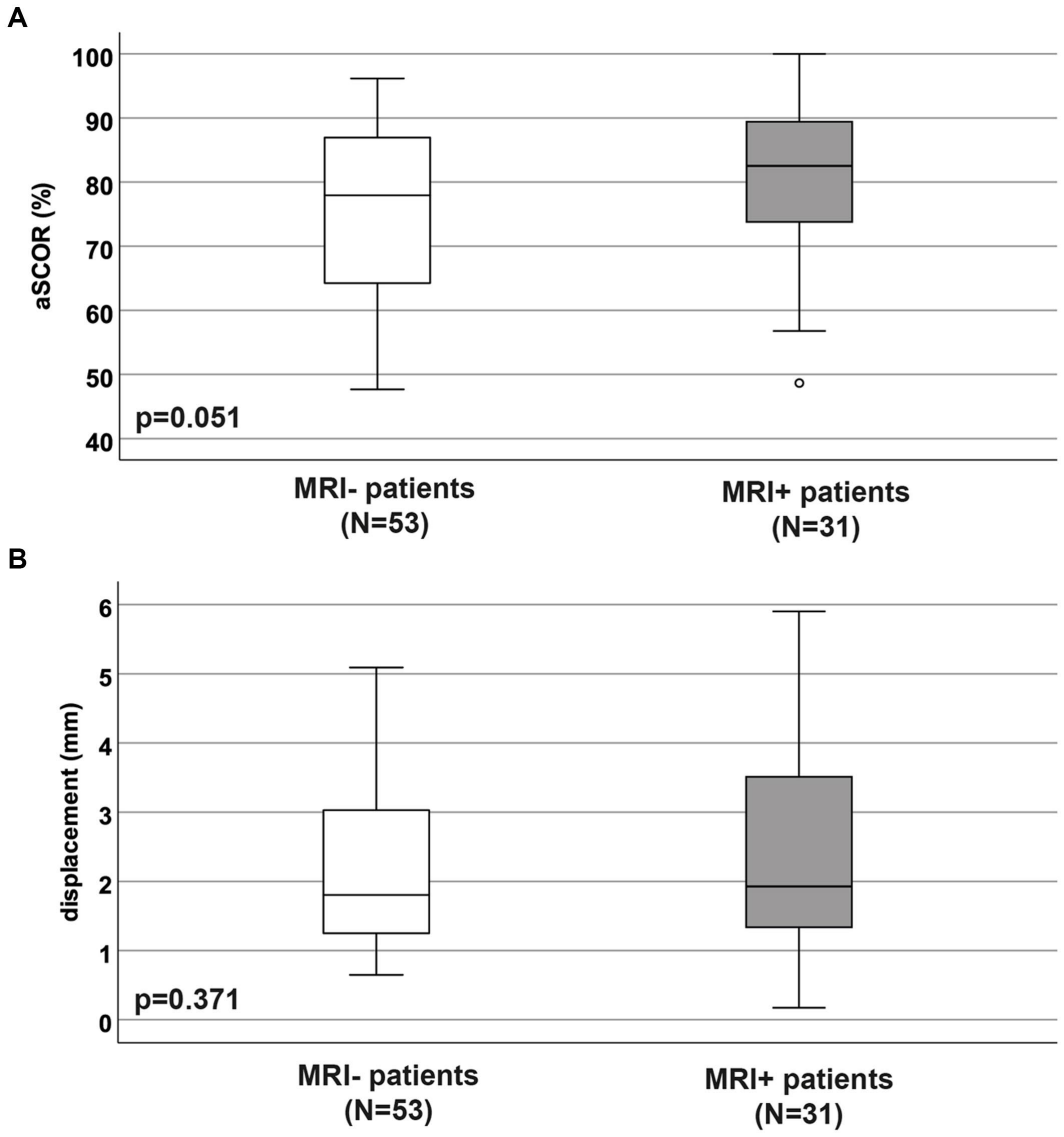
Adapted spinal canal occupation ratio and displacement values. The adapted spinal canal occupation ratio (**A**; aSCOR; %) and motion displacement values (**B**; mm) were not different between patient groups without (MRI−) and with (MRI+) T2w hyperintense lesion in MRI (*p* > 0.05).

While the proportions of the segmental location of the maximum stenosis differed between MRI+ and MRI− patients, maximum compression was most frequently located at segment C5/6 in both groups (Table 1D). The proportion of patients suffering from a multi-segmental cervical stenosis and the number of stenotic segments did not differ between the MRI+ and MRI− patients (Table 1D). Spinal canal constriction at the segment of maximum compression were comparable between the patient groups (aSCOR: MRI− patients: 77.9 [64.2–82.1] %; MRI+ patients: 82.5 [72.5–90.1] %; *p* = 0.051; Figure 2A).

### 3.5 Spinal cord motion values

Both, MRI+ and MRI− patients showed significantly increased spinal cord motion by means of displacement (Figure 2B). Displacement values did not differ between the MRI+ and MRI− groups (MRI− patients: 1.8 [1.2–3.0] mm; MRI+ patients: 1.9 [1.3–3.5] mm; *p* = 0.37; Figure 2B).

### 3.6 Diagnostic value of MRI parameter to identify patients with pathologic assessments

**Figure 3:**
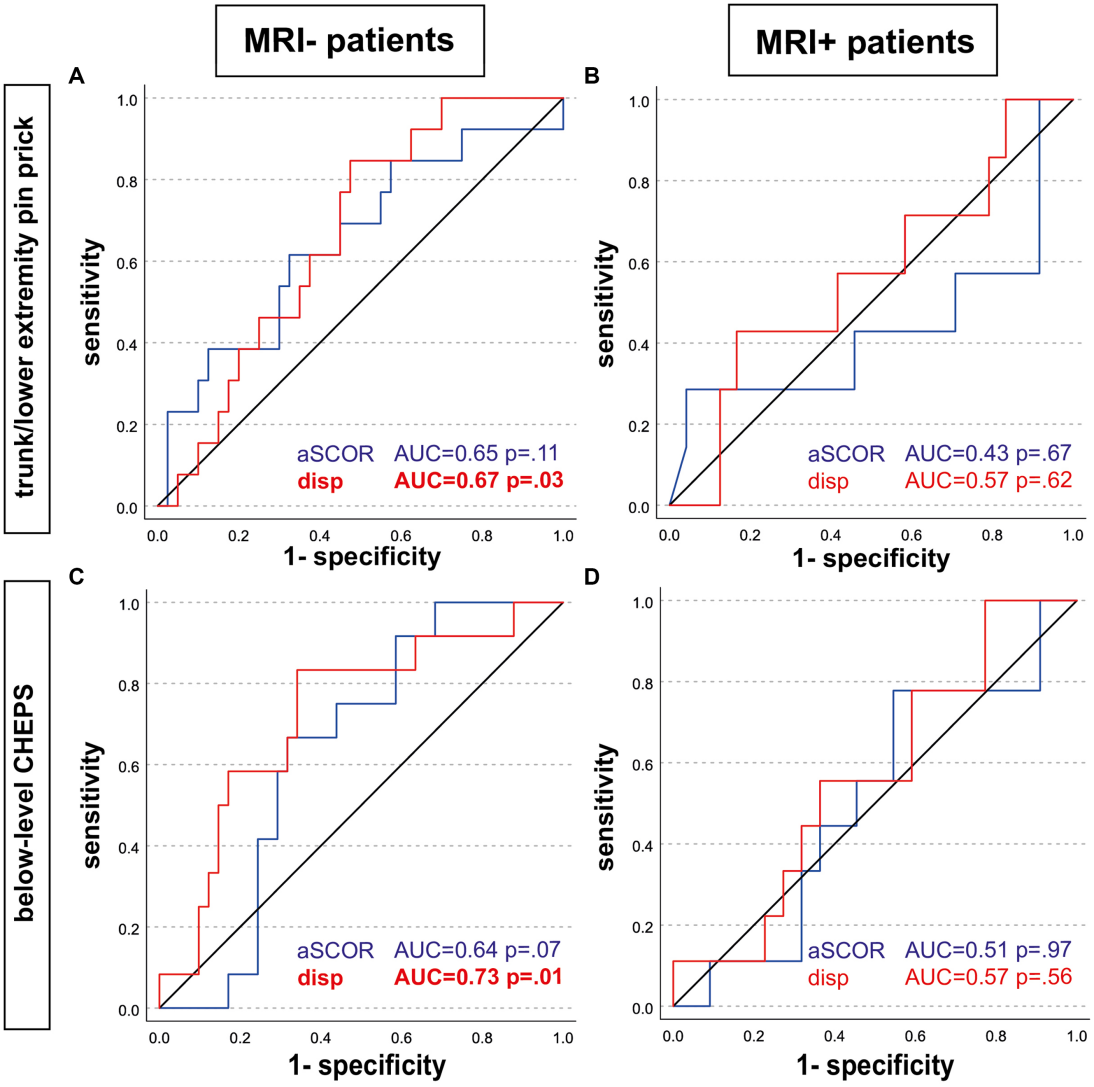
Receiver operating characteristic analysis. Receiver operating characteristic analysis curves are shown for patients without (left; MRI-; **A**,**C**) and with (right; MRI+; **B**,**D**) T2w hyperintense lesion in MRI. Predictors to identify patients with pathologic assessments were the adapted spinal canal occupation ratio (aSCOR; blue line) and the motion displacement values (disp; red line). Only in the MRI-group, displacement values were able to identify patients with pathologic trunk/lower extremity pin-prick score **(A)** and pathologic below-level CHEPS **(C)**. aSCOR, Adapted spinal canal occupation ratio; AUC, Area under curve; CHEPS, Contact heat evoked potentials; disp, Displacement.

Only in the MRI− group, spinal cord displacement values allowed to identify patients with pathological T/LEPP and pathological below-level CHEPS (Table 2; Figure 3). Most suitable displacement threshold values were 1.67 mm (sensitivity 84.6%, specificity 52.5%) for identification of MRI− patients with pathological T/LEPP and 1.96 mm (sensitivity 83.3%, specificity 65.6%) for identification of MR− patients with pathological below-level CHEPS. In the MRI+ group, displacement values did not identify patients with pathological assessments. The aSCOR values failed to identify patients with pathological assessments in both patient groups (*p* > 0.05; Table 2).

**Table 2:** Receiver operating characteristic analysis.

		MRI− patients				MRI+ patients			
		aSCOR		Displacement		aSCOR		Displacement	
		AUC	*p*	AUC	*p*	AUC	*p*	AUC	*p*
Upper extremities	Motor score	0.48	0.76	0.53	0.71	0.70	0.10	0.64	0.18
	Light touch score	0.48	0.82	0.61	0.18	0.51	0.94	0.62	0.24
	Monofilament score	0.50	1.00	0.51	0.87	0.54	0.82	0.50	1.00
	Pin prick score	0.50	0.97	0.59	0.28	0.56	0.55	0.55	0.66
	Segmental CHEPS	0.54	0.64	0.64	0.09	0.43	0.49	0.64	0.21
Trunk/lower extremities	Light touch score	0.52	0.88	0.60	0.30	0.35	0.26	0.54	0.74
	Pin prick score	0.65	0.11	**0.67**	**0.03**	0.43	0.67	0.57	0.62
	Below-level CHEPS	0.64	0.07	**0.73**	**0.01**	0.51	0.97	0.57	0.56

MRI−, Patients without T2w hyperintense lesion in MRI; MRI +, Patients with T2w hyperintense lesion in MRI; aSCOR, Adapted spinal canal occupation ratio; AUC, Area under curve; CHEPs, Contact heat evoked potentials.

## 4 Discussion

Patients with visible T2w hyperintense lesion (MRI+) and those without (MRI−) within the spinal cord presented with comparable extent of increased spinal cord motion (i.e., displacement) and spinal cord compression (i.e., aSCOR values). Only in the MRI− group, spinal cord displacement values were able to identify patients with pathologic clinical and neurophysiological assessments, while this was not true for MRI+ patients. Measures of locally exaggerated cord motion may therefore complement the diagnostic workup particularly in DCM patients with not yet established potentially irreversible spinal cord damage. Early detection of increased cord motion may enable proactive and timely surgical treatment in a disease stage preceding irreversible cord damage and clinical deterioration.

### 4.1 Clinical and neurological assessments in patients with and without T2w hyperintense lesion in MRI

While between 20 and 60% of DCM patients deteriorate over 3–6 years of follow up (31), the underlying pathophysiology is still poorly understood (7). Despite this notion and the fact that even extensive spinal stenosis poorly correlates to the clinical status (6, 32), decision for surgical treatment is often driven by the extent of compression and presence of a T2w hyperintensity observed in MRI (33). Importantly, T2w hyperintense lesions were found to be associated with poor postoperative outcomes (9), pointing to a potential irreversible spinal cord damage in this group. However, other studies did not affirm these findings, while postoperative improvements of spinal cord signal intensity changes were associated with superior outcomes (34). In conclusion, preoperative spinal cord signal intensity changes can reflect both, irreversible tissue damage and reversible alterations, mostly attributable to edema. However, there is a pressing need for additional biomarkers to effectively identify DCM patients to initiate surgical decompression prior to potential irreversible structural spinal cord damage. Most patients in our population presented with mild DCM (i.e., mJOA values >14), whereas previous cohorts evaluating surgical outcomes included more moderate (i.e., mJOA = 11–13) to severe (i.e., mJOA < 11) cases (35). In line with previous findings (27), pathologic results in the detailed neurological assessments were mostly observed by sensory examinations, while common motor assessments as applied here were normal in most patients. Most mild neurological impairment was rather related to spinothalamic tract (i.e., pin prick score) and dorsal column dysfunction (i.e., light touch/monofilament score). Interestingly, differences in clinical assessments between MRI− and MRI+ patient groups could be best revealed in mJOA subscores representing dexterity of upper extremities and gait, and in monofilament sensation of the hand dorsum. In line with our results, Berberat et al. (36) also reported no differences for the total mJOA between patients with and without T2w hyperintense lesions. Gait, upper limb numbness, and impaired dexterity were previously reported as early symptoms in DCM, with accentuated deficits in more severe cases (37, 38).

### 4.2 Association of increased motion to pathologic assessments

Spinal cord damage and neurological deterioration in DCM is assumed to be caused by compression and repetitive dynamic microtrauma to the cord (39). Mechanical forces to the cord cause a direct injury on neuronal and glial cells and trigger a cascade of pathobiological processes (i.e., ischemia, neuroinflammation, and apoptosis) (39). We hypothesize, that pathologically increased spinal cord motion causes intensified dynamic mechanical strain as part of the DCM pathophysiology and subsequently adds to spinal cord tissue damage. In line with this hypothesis previous work revealed, that increased spinal cord motion at a focal stenosis induces mechanical strain on the entire spinal cord due to stretch and compression of adjacent cord tissue (19). The kinetic energy of spinal cord tissue increases with the square of its velocity value (E = m*v^2^; E = energy; m = mass; v = velocity), suggesting increased dynamic mechanical strain with increasing velocities during oscillations. In a bovine model could be demonstrated that mechanical strain parallel to white matter fiber direction (i.e., tissue stretch) increased cord tissue stress and stress levels increased with higher velocities of external forces (40). Recently, a computational model additionally demonstrated mechanical strain to the spinal cord caused by cord motion/oscillations comparable to a dynamic compression model during spine flexion and extension (20). Importantly, spinal cord oscillations (i.e., 60–80 times per minute) occur much more frequently compared to neck movements. Of note, only in MRI− patients cord displacement values were related to neurological and neurophysiological signs of spinothalamic tract dysfunction (i.e., pin prick sensation of the trunk and lower extremities; below-level contact heat evoked potentials representing integrity of the spinothalamic tract), despite the lack of differences in spinal cord motion (i.e., displacement values) between MRI+ and MRI− patient groups. As T2w hyperintense signal in the spinal cord reflect diverse underlying pathologies (i.e., reversible edema vs. irreversible tissue scarring), we hypothesized that alterations in spinal cord tissue properties might contribute to the observed group differences. These varied pathologies associated with T2-weighted hyperintense signals could affect the dynamics of cord motion, potentially diminishing their diagnostic value as biomarkers in MRI+ patients. Underlining this hypothesis, several microstructural MRI studies demonstrated tissue changes in DCM patients indicating demyelination and axonal loss [e.g., (41, 42)], while post-mortem studies confirmed histopathological changes (i.e., extensive white and gray matter degeneration, cavity formation, and loss of neurons and axons) (43). Remote neurodegeneration in the spinal cord and even in the brain was recently shown to be more pronounced in patients with T2w hyperintense signal changes within the spinal cord (44). Assuming that dynamic mechanical strain contributes to the development of DCM, MRI+ patients may have already been subject to increased motion for a longer time or the severity of the tissue injury may have occurred in a shorter amount of time (i.e., severity of injury independent of time). Symptom duration was not documented in our study cohort, while a distinct symptom onset in DCM is often hard to determine. Within the DCM pathophysiology different mechanism may predominantly cause spinal cord tissue damage and neurological deterioration. While in patients with less spinal canal constriction dynamic strain caused by increased cord motion may be of higher importance, static cord compression could be the main impact in patients with higher grade stenosis. However, measures of spinal canal constriction (i.e., aSCOR) did not identify patients with worse assessments in both groups in our population. Previous studies showed no or only modest correlations of anatomic readouts to the patient’s functional status (6, 11, 12, 32). These discrepancies may be attributed to the varying methods used for functional patient assessments and the different anatomical readouts utilized in the analyses. Some functional measurements, such as detailed sensory assessments, may be more sensitive to functional loss compared to more general assessments like the mJOA score. Additionally, the anatomical readouts may reflect spinal canal narrowing in different ways, contributing to the inconsistencies. Currently, there is no established gold standard for anatomical readouts to characterize spinal stenosis. In line with our results, an association of increased spinal cord motion to worse neurological (15, 17) and neurophysiological findings (16) was reported. In addition, we demonstrated, that spinal cord motion measures are superior to measures of the severity of the stenosis, particularly in identifying MRI− patients with pathologic neurological and neurophysiological assessments. While thresholds of critically increased cord motion could be obtained in our analysis, they have to be applied cautiously and cannot be generalized. A comparison across centers evaluating spinal cord motion in DCM patients has shown substantial differences of obtained cord motion readouts (45), underling the need of further standardizations of these measurements.

### 4.3 Clinical significance of cord motion measurements

**Figure 4:**
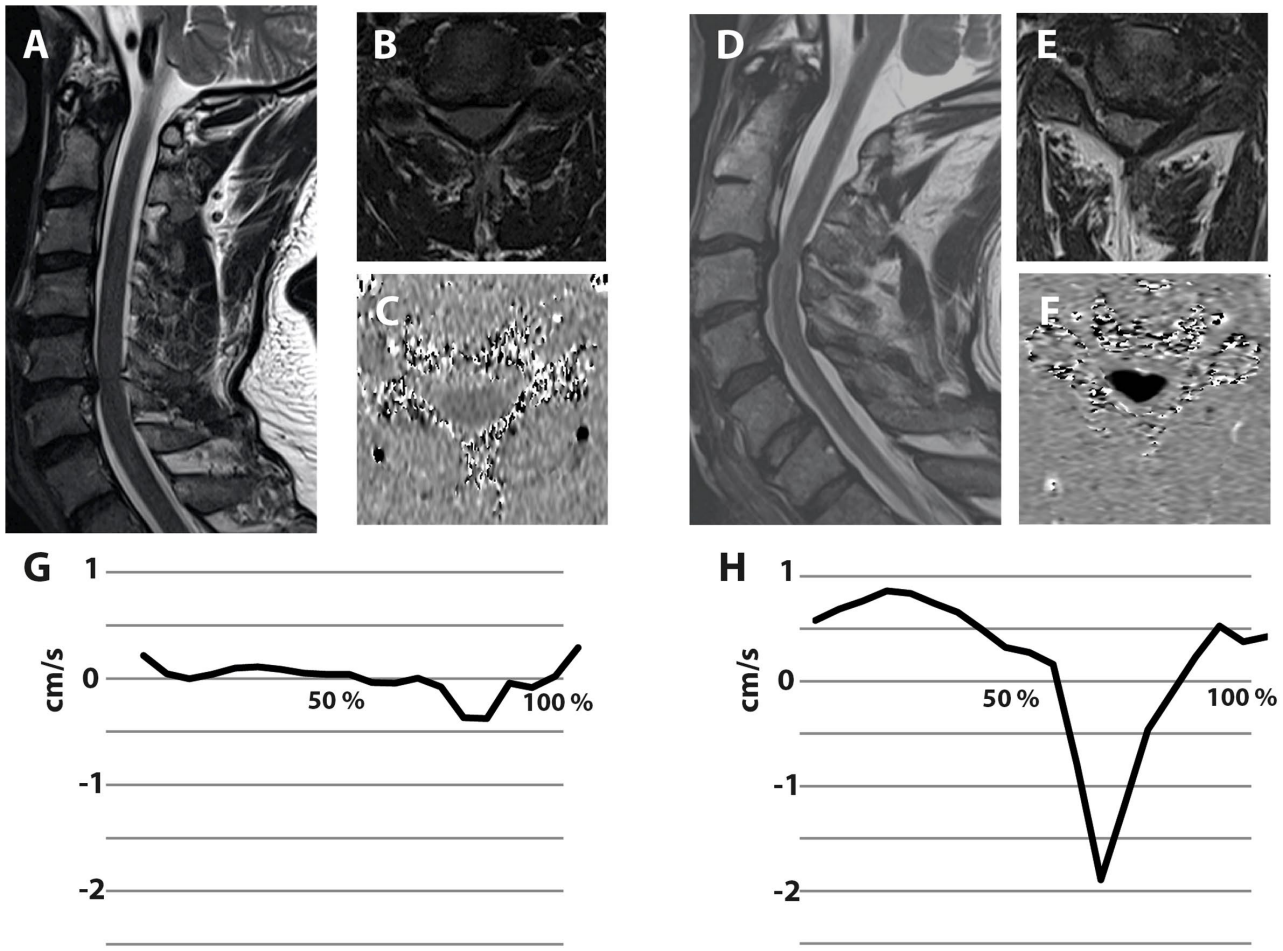
Illustrative examples of two patients suffering from degenerative cervical myelopathy with different spinal cord motion pattern despite comparable extent of spinal canal constriction. Both illustrated patients are suffering from degenerative cervical myelopathy. The patient on the left side presented with a cervical stenosis at the segment C5/6, while the stenosis of the patient on the right side is located at C3/4 (**A**,**D**—Sagittal T2-w; **B**,**E**—axial T2-w at the stenotic segment). Axial phase contrast MRI was collected at the stenotic segment **(C,F)**, while darker colors encode higher caudal velocities. The spinal cord motion velocity pattern (*y*-axis; cm/s) is plotted during the cardiac cycle (*x*-axis; %)—negative values represent caudal motion, positive values cranial motion **(G,H)**. The extent of spinal canal constriction appears comparable in both patients. Interestingly, the patient on the right presents with extensively increased spinal cord motion **(H)**, while the patient on the left shows a spinal cord motion pattern **(G)** comparable to healthy controls.

Currently, the clinical workup of DCM is based on clinical and neurophysiological assessments, as well as static MRI. These assessments reveal the present symptom burden (i.e., extent of spinal cord damage), but limited information on the further disease course. In contrast, spinal cord motion measurements hold the potential to complement information on the dynamic mechanical strain onto the spinal cord. Patients with a comparable anatomic situation (i.e., extent of stenosis) can present with divergent motion pattern (Figure 4), pointing further to its potential as an additional biomarker of mechanical cord strain. Importantly, increased spinal cord oscillations (i.e., displacement values) can already be observed in mild DCM patients. Motion measurements could help to particularly identify patients at risk for disease progression preceding irreversible tissue damage, while this has to be demonstrated in prospective analysis.

### 4.4 Limitations

Our study has several limitations. A smaller number of MRI+ patients compared to MRI− patients may reduce the power of the statistical analysis in this group. In MRI+ patients T2w hyperintensity can reflect both, irreversible tissue scar and reversible edema. Advanced macro- and microstructural MRI modalities may help to determine the severity of spinal cord damage, particularly in MRI+ patients. We did not correct for multiple comparisons as the study focuses on only a few scientifically sensible comparisons, rather than every possible comparison (i.e., planned comparison of spinal cord motion and anatomic information to the patient’s assessments). The statistical analysis was based on the hypothesis that higher motion (i.e., higher displacement values) and higher-grade stenosis (i.e., higher aSCOR values) relate to pathologic assessments in patients. Spinal cord motion was consistently related to signs of spinothalamic tract damage (i.e., worse pin-prick sensation and worse electrophysiological measurements of the spinothalamic tract). While the outcomes are in ordinal scale, they had to be dichotomized for the ROC analysis. The severity of the impairment was not considered in the analysis, but most patients suffered only from mild impairment. Therefor the risk of misinterpretation should be negligible. While we only assessed relations of spinal cord motion to neurological and neurophysiological findings in a cross-sectional analysis, the predictive value of spinal cord motion measurements to identify patients at risk for disease progression will benefit from longitudinal studies. Additionally, defining rigorous thresholds of critically increased spinal cord motion as means of potentially detrimental impact toward the spinal cord requires larger population-based studies to be performed. Further studies are needed to prove obtained thresholds of spinal cord motion across different evaluation techniques and measurement sites. Potential confounders of spinal cord motion measurements (e.g., breathing; blood pressure) were not considered in this analysis.

### 4.5 Conclusion and future direction

In conclusion, increased spinal cord motion has been revealed as an underrecognized component of the DCM pathophysiology, suggesting that increased cord motion contributes to dynamic mechanical strain, and consequently to spinal cord tissue damage. We propose that spinal cord motion assessment can complement diagnostic workups specifically in early/mild DCM myelopathy, as treatment decisions in these patients are in need of quantifiable measures for objective assessments. Prospective studies are warranted to validate spinal cord motion threshold values (obtained in this cross-sectional analysis) to identify patients at risk for disease progression, who should be assigned to surgical decompression.

## Data availability statement

The raw data supporting the conclusions of this article will be made available by the authors, without undue reservation.

## Ethics statement

The studies involving humans were approved by Kantonale Ethikkommission Zurich, KEK-ZH 2012-0343, BASEC Nr. PB_2016-00623. The studies were conducted in accordance with the local legislation and institutional requirements. The participants provided their written informed consent to participate in this study.

## Author contributions

NP: Data curation, Funding acquisition, Investigation, Project administration, Resources, Writing – review & editing. CJ: Formal analysis, Writing – review & editing. MiH: Data curation, Writing – review & editing. PS: Data curation, Visualization, Writing – review & editing. DP: Formal analysis, Writing – review & editing. CZ: Investigation, Project administration, Resources, Writing – review & editing. JR: Data curation, Investigation, Resources, Writing – review & editing, Validation. SF: Investigation, Resources, Writing – review & editing. RS: Resources, Writing – review & editing. JS: Funding acquisition, Investigation, Resources, Writing – review & editing. MB: Investigation, Resources, Writing – review & editing. MSc: Funding acquisition, Investigation, Resources, Writing – review & editing. MSe: Writing – review & editing. PF: Funding acquisition, Investigation, Resources, Supervision, Writing – review & editing. MF: Funding acquisition, Investigation, Methodology, Resources, Supervision, Writing – review & editing. AC: Funding acquisition, Investigation, Methodology, Resources, Supervision, Writing – review & editing. MaH: Data curation, Formal analysis, Funding acquisition, Investigation, Methodology, Project administration, Resources, Visualization, Writing – original draft.

## Footnotes

^1^ www.horosproject.org
